# Estimating the clinical and financial burden of motorcycle injuries on a rural faith-based hospital in Kenya: a 9-month retrospective review

**DOI:** 10.11604/pamj.2026.53.89.50174

**Published:** 2026-02-17

**Authors:** Isaac Batian Mikanatha, Ntonja Kenneth Gitonga, Griffins Omondi, Eric Muthuri Mibuari, Kathleen Glenda Julian

**Affiliations:** 1 Whiting School of Engineering, Johns Hopkins University, Baltimore, Maryland, United States,; 2 Information Technology, Maua Methodist Hospital, Maua, Kenya,; 3 Office of Medical Director, Maua Methodist Hospital, Maua, Kenya,; 4 School of Engineering and Applied Sciences, Harvard University, Cambridge, Massachusetts, United States,; 5 Department of Medicine, Penn State Health Hershey Medical Center, Penn State, Hershey, United States

**Keywords:** Motorcycles, wounds and injuries, accidents, traffic, health care costs, Kenya, Africa

## Abstract

As motorcycle use increases across Africa, hospitals face rising clinical demands to treat injuries from motorcycle crashes. This study assesses the burden of motorcycle-related injuries on a faith-based hospital in rural Kenya, focusing on unreimbursed care. A retrospective review was conducted of patients presenting for care from September 2023 to May 2024 at Maua Methodist Hospital. Case-finding utilized International Classification of Diseases-10 (ICD-10) injury codes and the handwritten Casualty Department log; patient encounters were included in the analysis if clinical documentation confirmed a motorcycle injury. Of the 225 patients identified who presented for motorcycle-related injuries, the median age was 32 years, 35 (16%) were children, 20 (9%) were aged ≥65 years, and 18% (17 of 95 in the Casualty Department log) were pedestrians hit by motorcycles. Thirty-one percent (29 of 95) of patients who presented to the Casualty Department were referred for higher-level care, primarily for head injuries. Four patients died on presentation. Of the 23 hospitalizations, 15 (65%) included surgery, the primary driver of costs. Fifty-two percent of the 290 patient encounters were uninsured; reimbursements were especially poor for hospitalizations. In conclusion, multiple persons with motorcycle injuries presented each week for care at this faith-based hospital, incurring >1,755,000 KES (>13,500 USD) in unreimbursed charges over the nine months. Due to limitations in documentation and differences in healthcare costs between countries, this underestimates the burden of motorcycle-related injuries. Improving ICD-10-coded data for traffic accident injuries, as has been utilized by some trauma registries in Africa, can help monitor cases and costs, and advocate for motorcycle safety.

## Introduction

With increased availability in Kenya and other African countries [1], motorcycles have become a major mode of transportation. From 2017 to 2021, the number of registered motorcycle operators in Kenya nearly doubled [2]. In rural areas, motorcycles are popular as taxis due to low cost and excellent maneuverability on unpaved paths. However, it is not rare for motorcycles to be overloaded or to fail to follow basic traffic rules-examples of preventable risk factors for collisions [3]. Between May 2022 and October 2023, the Kenyan Police reported ~10,000 motorcycle crashes, including 2,384 fatalities [2]. To assist persons injured in motorcycle crashes, there is an increased demand on healthcare systems to provide emergency and post-trauma care [4]. The purpose of this study was to describe persons presenting for care of motorcycle-related injuries and to assess the associated financial burden of non-reimbursed care at a faith-based hospital in rural Kenya, Maua Methodist Hospital (MMH). International Classification of Diseases-10 diagnosis (ICD-10) codes were evaluated as a data-capture method for tracking injured patients for future surveillance of motorcycle traumas. Data on the impact of motorcycle injuries may be useful to advocate for hospital support and to promote motorcycle safety policies.

## Methods

**Setting:** as a rural 200-bed level-4 hospital, MMH provides ambulatory, emergency, and hospital care, inclusive of orthopedic and general surgery, for residents of Igembe and Tigania subcounties of Meru County. While many road traffic injuries can be treated at MMH, it did not have a computed tomography (CT) scanner; severe head and intra-abdominal injuries are referred to level-5 hospitals (e.g., Meru Teaching and Referral Hospital (distance, 50 km)).

**Data sources:** Maua Methodist Hospital maintains an electronic database that records each outpatient and inpatient encounter, uniquely identified by name, visit number, and registration number. A primary diagnosis field, required to be completed by staff for each encounter, contains an ICD-10 diagnosis code. In early 2023, MMH began using cause-of-injury ICD-10 codes. In the casualty and outpatient departments, clinicians wrote clinical notes in an electronic medical record. For further characterizations of injuries, data were obtained by manual review of clinician notes in the electronic medical record and of a hand-written log in the Casualty Department, where staff described patients presenting after road-traffic accidents. Charges and reimbursements were extracted from MMH electronic financial databases; actual costs were not available. Unpaid bills were calculated by subtracting patient reimbursement (cash or insurance) from the hospital charge.

**Study design:** to identify persons treated for motorcycle-related injuries, a retrospective chart review was conducted of outpatients and inpatients presenting to MMH from September 2023 to May 2024. International Classification of Diseases-10 codes in the “primary diagnosis” field of an electronic report were utilized to screen cases. To identify additional cases not captured by ICD-10 codes, each encounter in the handwritten Casualty Department log was reviewed.

**Participants-inpatients:** inpatient encounters with transport-related ICD-10 codes (V-series) or general injury codes (S- or T-series) were included if the clinical notes in the electronic medical record confirmed a motorcycle as the cause of injury.

**Participants-outpatients:** for the Casualty Department, encounters with transport-specific ICD-10 (V-series) codes and/or entries in the handwritten Casualty Department log were included if notes confirmed a motorcycle injury. Non-Casualty Department outpatient encounters were identified by motorcycle-specific ICD-10 codes (V20-V29). Due to the large number of outpatient encounters, it was not possible to conduct a manual review of outpatients with S- or T-codes.

**Use of ICD-10 codes for surveillance of motorcycle injuries:** to guide future surveillance, clinician coding patterns were assessed for the “primary diagnosis” applied to inpatient encounters and to encounters in the handwritten Casualty Department log.

**Variables:** data collected for the study included demographics, ICD-10 codes, pedestrian vs motorcycle rider, referral to higher-level care, type of injury, hospitalization, surgery, insurance status, charges, and reimbursement.

**Bias:** despite efforts to comprehensively capture cases, coding and documentation issues led to under-estimation of cases. Cases could be missed if coded nonspecifically (e.g., M- codes for musculoskeletal pain) or if clinical documentation only notated “RTA” (road traffic accident) without mentioning a motorcycle; while some of these were likely motorcycle-related, unconfirmed cases were not included.

**Study size:** this was determined by feasibility limits of the study timeframe, including when ICD-10 coding implementation was advanced.

**Statistical methods:** given the descriptive nature of the study, sampling methods were not needed, and no comparisons were conducted that would require statistical tests. In regards to missing data, two variables (motorcycle-injury patients who were identified as pedestrians, and patients requiring transfer to a higher level of care) were only available in the handwritten casualty log. Denominators were included to account for these limited subsets.

**Ethical approval:** this study was approved by the MMH Institutional Review and Ethics Board, reference number: MMH-IREB/Approval/2024/02.

## Results

**Patient characteristics:** during the 9-month study period, 267 outpatient encounters (including 95 in the Casualty Department) and 23 inpatient encounters were confirmed to be related to injuries from motorcycle crashes. For the 225 individuals with inpatient and/or outpatient encounters for motorcycle-related injuries, the median age was 32 years (range, 2-84 years), and 150 (67%) were males. Vulnerable subgroups included 35 (16%) children (aged ≤17 years) and 20 (9%) who were aged ≥65 years. Injuries documented in the Casualty Department resulted primarily from persons riding motorcycles during collisions; 18% (17 of 95 in the Casualty Department log) were pedestrians hit by motorcycles. Four people (including two pedestrians) were brought in as bodies or died of injuries in the Casualty Department. Thirty-one percent (29 of 95) of patients presenting to the Casualty Department for injuries were referred/transferred to other hospitals with CT scanners, most commonly for head injuries. Patients hospitalized at MMH were primarily treated for leg injuries; 15 (65%) of 23 inpatients underwent surgery (generally for orthopedic procedures and/or wound debridement). The median length of hospitalization was 4 days (range, 1-14 days). Fifty-two percent of the 290 patient encounters were uninsured.

**Inpatient unreimbursed charges:** during the 9-month study period, 1,538,900 KES (11,800 USD) (median per hospitalization, 45,000 KES (340 USD)) in charges were identified for care of persons injured in motorcycle crashes. Services, which included primarily surgical procedures, comprised 48% of total charges; pharmacy (27%) and lab (7%) were other components. While 74% of 23 inpatient encounters were insured, insurance only reimbursed MMH for 29% of these charges. Maua Methodist Hospital (MMH) absorbed 99% of the bills for uninsured inpatients. Overall, unpaid charges were 1,167,200 KES (9,000 USD) (median, 32,100 KES (245 USD) per inpatient encounter).

**Outpatient/Casualty Department unreimbursed charges:** for the 267 outpatient encounters identified as receiving care for motorcycle injuries, unreimbursed charges were >587,284 KES (>4,500 USD) for the 9-month study period (median per outpatient encounter, 1,476 KES (11 USD).

**Assessment of ICD-10 codes for surveillance of motorcycle injuries:** it became apparent during the study that clinicians usually coded the type of injury (e.g., S72.0 for femur fracture) instead of the cause of injury (transport-specific V-codes). Only 8 inpatient encounters during the study period had V-codes assigned as the primary diagnosis for the inpatient encounter or for the day-of-admission Casualty Department encounter; six of these were confirmed to be motorcycle-related. Of the 144 inpatient encounters during the study period that had general injury S- or T-codes assigned as the primary diagnosis, 10% of these were confirmed to be due to motorcycle injuries. Of the 95 encounters documented in the Casualty Department logs as motorcycle injuries, 21 (22%) had a motorcycle-specific injury V-code as the primary diagnosis.

## Discussion

The rapid increase in motorcycle use has brought unprecedented mobility and economic opportunities to rural Kenya; it has also resulted in substantial human costs, including injuries and strain on healthcare facilities. During the nine months of this study at MMH, a level-4 hospital, multiple persons with motorcycle injuries presented each week to the Casualty Department (including 4 who died on presentation); >30% of injured persons required referral/transfer to hospitals with CT scanners, primarily for head injuries. While 23 hospitalizations were confirmed at MMH, many more patients presented for outpatient clinic care (>267 encounters). As will be discussed, outpatient encounters were especially difficult to capture and were greatly underestimated in this study.

To our knowledge, this paper presents one of the few estimates of the clinical and financial burden of motorcycle injuries in a rural faith-based hospital in Kenya. For inpatients alone, unpaid charges exceeded ≥1,167,000 KES (>9,000 USD). Most motorcycle-injury hospitalizations involved surgeries. This was the primary driver of treatment costs, followed by pharmacy. A study at trauma hospitals in Kisumu, Kenya (including one level-6 and two level-5 hospitals), found that surgery accounted for almost half of hospital costs for motorcycle injuries [5]. In that different, urban setting, it is striking that 53% of all major surgeries were performed for motorcycle injuries.

At MMH, the financial burden for care of motorcycle-related injuries was in part because half of the patients had no insurance. At this faith-based hospital, uninsured patients paid for <1% of hospitalization charges (median per hospitalization, 45,000 KES (340 USD), which is equivalent to 2-3 months of a boda-boda operator´s income [6]). Other authors have proposed that all motorcycle operators should have healthcare insurance (e.g., the National Health Insurance Fund) [4]. As alluded to in Kenyan regulations, this insurance should also cover passengers and injured pedestrians.

It is acknowledged that this analysis included charges (not actual costs) and that charges vary significantly by different hospitals; however, MMH follows the Kenyan Ministry of Health guidelines for charges, which were extremely low relative to other countries. Thus, “low” costs can be misleading-all unreimbursed care is detrimental to a faith-based hospital with minimal avenues for income. Secondly, financial scales differ widely between countries. The Gross Domestic Product per capita (purchasing power parity) in the United States is more than ten-times higher than in Kenya [7]. One study estimated the adjusted direct medical costs for a femur fracture treated in the United States to be 18,000 USD for a single patient [8].

This study also evaluated the role of ICD-10-coded clinical data for surveillance of motorcycle injuries. In this early stage of implementation, only 22% of Casualty Department encounters for motorcycle injuries had a motorcycle-specific injury code. It was also difficult to capture clinic encounters for post-trauma care (e.g., pain syndromes) where the etiology of injury (e.g., transport-specific injury codes) was not used. To better utilize ICD-10 codes for trauma surveillance, components could be strengthened, including: 1) clinician-oriented data-entry interfaces with required fields for coding the cause of injury, and 2) reports that comprehensively summarize ICD-10-coded data. Compared to resource-intensive studies with researchers collecting prospective data [5], ICD-10-coded data has the potential to be an efficient data-capture method for facilitating trauma surveillance.

Limitations of this study include the under-representation of the burden of motorcycle injuries in rural Kenya. This study cannot capture the long-term sequelae of injuries, demands on clinicians, and diversion of resources from other healthcare needs.

## Conclusion

Motorcycle injuries in rural Kenya caused mortality, morbidity, and financial burden at this faith-based hospital. While our study captures part of the impact, it was a significant underestimate. As stated by other authors [9], authorities will not prioritize transportation safety or support for hospitals if the scale of motorcycle injuries is not adequately measured and communicated. With improvements in ICD-10 coding infrastructure (e.g., required data fields to designate transport-associated injuries), hospitals can efficiently enhance surveillance to better measure the human and financial burden of motorcycle injuries-this has been utilized by other trauma registries in Africa [10]. Sharing of comprehensive data about injuries can help advocate for reimbursement for hospitals, broadened healthcare insurance coverage, and policy measures to improve transportation safety.
